# Altered attentional control over the salience network in complex regional pain syndrome

**DOI:** 10.1038/s41598-018-25757-2

**Published:** 2018-05-10

**Authors:** Jungyoon Kim, Ilhyang Kang, Yong-An Chung, Tae-Suk Kim, Eun Namgung, Suji Lee, Jin Kyoung Oh, Hyeonseok S. Jeong, Hanbyul Cho, Myeong Ju Kim, Tammy D. Kim, Soo Hyun Choi, Soo Mee Lim, In Kyoon Lyoo, Sujung Yoon

**Affiliations:** 1 Ewha Brain Institute, Ewha Womnans University, Seoul, South Korea; 2 Department of Brain and Cognitive Sciences, Ewha Womans University, Seoul, South Korea; 3 Department of Radiology, Incheon St. Mary’s Hospital, The Catholic University of Korea College of Medicine, Seoul, South Korea; 4 Department of Psychiatry, The Catholic University of Korea College of Medicine, Seoul, South Korea; 5 School of Science and Engineering, Tulane University, New Orleans, USA; 6 Department of Radiology, Ewha Womans University College of Medicine, Seoul, South Korea; 7 College of Pharmacy, Graduate School of Pharmaceutical Sciences, Ewha Womans University, Seoul, South Korea

## Abstract

The degree and salience of pain have been known to be constantly monitored and modulated by the brain. In the case of maladaptive neural responses as reported in centralized pain conditions such as complex regional pain syndrome (CRPS), the perception of pain is amplified and remains elevated even without sustained peripheral pain inputs. Given that the attentional state of the brain greatly influences the perception and interpretation of pain, we investigated the role of the attention network and its dynamic interactions with other pain-related networks of the brain in CRPS. We examined alterations in the intra- and inter-network functional connectivities in 21 individuals with CRPS and 49 controls. CRPS-related reduction in intra-network functional connectivity was found in the attention network. Individuals with CRPS had greater inter-network connectivities between the attention and salience networks as compared with healthy controls. Furthermore, individuals within the CRPS group with high levels of pain catastrophizing showed greater inter-network connectivities between the attention and salience networks. Taken together, the current findings suggest that these altered connectivities may be potentially associated with the maladaptive pain coping as found in CRPS patients.

## Introduction

Chronic pain is one of the major public health problems due to its debilitating effects on quality of life and function^1–3^. Along with a negative emotional state and feelings of helplessness, the amplification of pain intensity and continued pain-related rumination are frequently observed symptoms in individuals with chronic pain^4^. Central sensitization or centralization of pain, a condition of increased neural signaling in the central nervous system that generates hypersensitivity to pain^5^, may be linked to structural or functional changes in the brain^6–8^. This condition has been suggested to play a role in the pathophysiological mechanisms underlying chronic pain^5,9,10^. There is growing interest in further investigating these pathophysiological mechanisms regarding centralized pain^11^. Furthermore, recent technological advances in noninvasive neuroimaging and network analysis have expanded our understanding of the dynamic and interactive role of the brain networks in centralized pain conditions^12^.

Perception and interpretation of pain may be substantially dependent on the attentional state of the brain^12,13^. Studies have shown that explicit manipulation of the attentional state may influence the perception and neural processing of pain^14–16^, and in the same way, experience of pain may also alter cognitive functions including attention^17,18^.

Pain is a rather attention-demanding and salient stimuli amongst other somatosensory modalities. As such, the salience network of the brain has received considerable interest in research regarding centralized pain, due to its activation when attending to painful stimuli^12^. Studies have reported both functional and structural abnormalities of the salience network in individuals with centralized pain conditions^11,18–20^. In addition, the attention and salience networks have been suggested to be closely associated with each other, as saliency detection is often dependent on attentional processes^21,22^.

Alterations were also found in other resting-state networks (RSNs) including the default mode network (DMN) and somatosensory network in individuals with centralized pain conditions^12,20,23–26^. However, there remains a dearth of information regarding the dynamic interactions between the attention network and aforementioned pain-related RSNs in individuals with centralized pain conditions.

The aim of the current study is to characterize the alterations associated with centralized pain in intra-network functional connectivities of pain-related RSNs including the DMN, salience, and sensorimotor networks. We employed resting-state functional magnetic resonance imaging (fMRI) to investigate 21 patients with complex regional pain syndrome (CRPS), a condition which has been recognized as a classic example of centralized pain conditions^5^, and 49 healthy individuals who were matched for age and sex. We also investigated possible alterations of inter-network functional connectivities between the attention network and other pain-related RSNs in individuals with CRPS, as well as its relationship with the level of pain catastrophizing, using the Pain Catastrophizing Scale (PCS)^27^.

## Results

### Differences in Functional Connectivity within the Pain-related and Attention Networks

**Figure 1:**
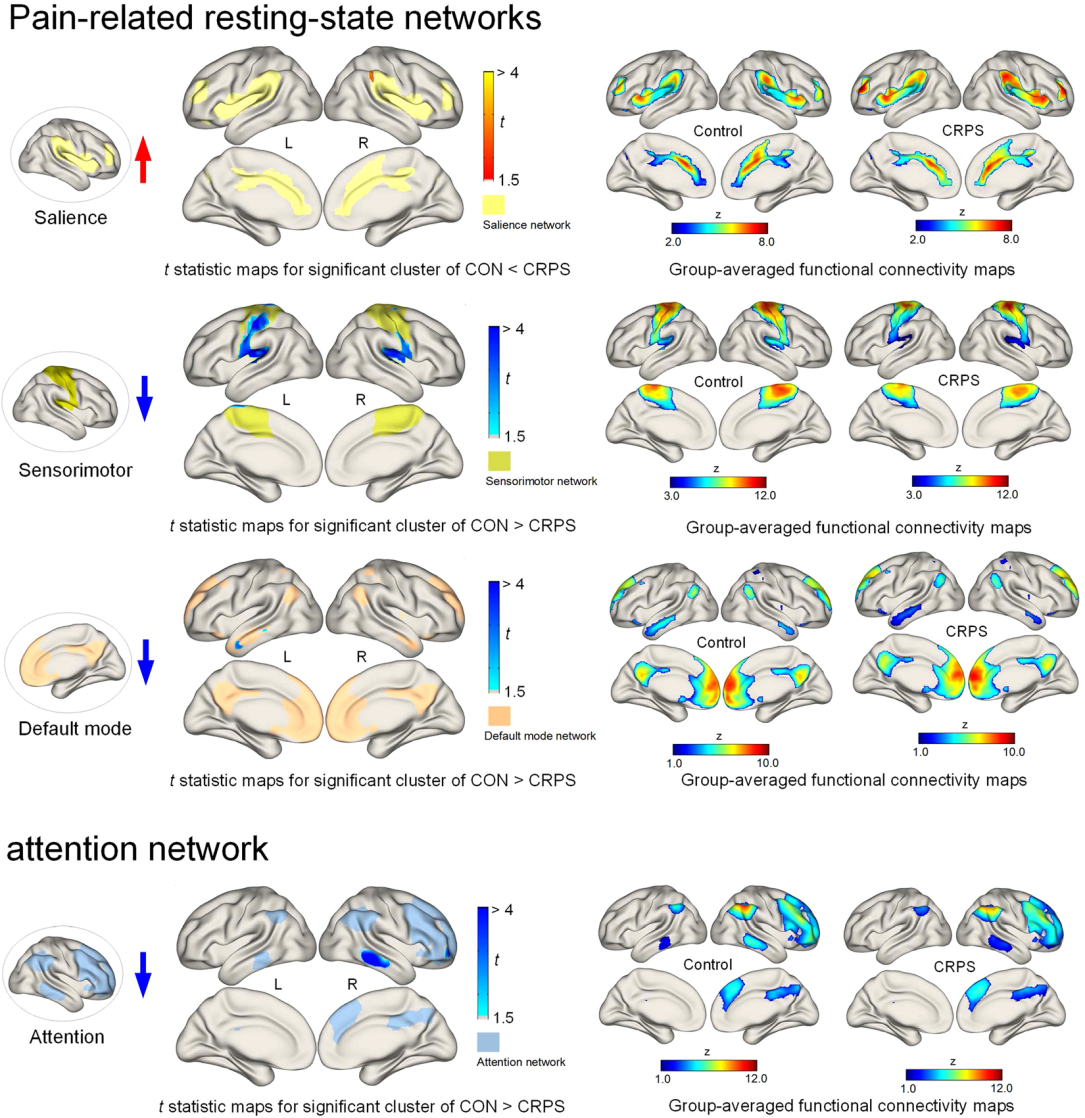
Statistical parametric map of the *t*-statistic images of significant clusters indicating the group differences in functional connectivity and group-averaged functional connectivity maps for each group (control vs. CRPS). For clusters in the attention network, there were reduced functional connectivity in individuals with CRPS relative to the control group. For the pain-related RSNs, individuals with CRPS showed enhanced functional connectivity in the salience network as compared with the control group. In contrast, reduced functional connectivity was observed in the clusters of the sensorimotor and default mode networks in the CRPS group, as compared with the control group. BrainNet Viewer^50^ was used to visualize three-dimensional rendering of the clusters and the RSNs in the MNI space. CON, control; CRPS, complex regional pain syndrome; RSN, resting state network; MNI, Montreal Neurological Institute.

A significant cluster of enhanced functional connectivity was found in the salience network of individuals with CRPS relative to that of healthy individuals at corrected *P* < 0.05 (Fig. 1 and Table 1). In contrast to this enhancement, individuals with CRPS had clusters of significantly reduced functional connectivity in other pain-related RSNs including the sensorimotor and default mode networks at corrected *P* < 0.05, as compared with healthy individuals (Fig. 1 and Table 1). We also found clusters of reduced functional connectivity in the attention network in individuals with CRPS relative to healthy individuals (Fig. 1 and Table 1).

**Table 1:** Cluster information of voxel-wise functional connectivity alterations related to CRPS.

Network	Anatomical location	Cluster size (mm^3^)	Maximum *t* value	MNI atlas coordinates		
				(location of maximum t-value)		
				*x*	*y*	*z*
**Enhanced functional connectivity in the CRPS group relative to the control group**						
Salience	Temporoparietal junction (R)	1,216	3.78	50	−46	40
**Reduced functional connectivity in the CRPS group relative to the control group**						
Attention	Inferior temporal gyrus (R)	10,432	5.70	62	−30	−20
	Frontal pole (R)	1,856	5.64	42	62	−8
Sensorimotor	Central opercular cortex (L)	35,136	6.40	−42	−14	16
	Planum temporale (R)	24,320	5.91	58	−18	8
	Postcentral gyrus (L)	768	4.93	−14	−34	80
	Precentral gyrus (R)	512	4.55	26	−14	76
Default mode (Anterior)	Middle temporal gyrus (L)	384	3.71	−66	−30	−16
	Middle temporal gyrus (L)	192	3.67	−66	−6	−16
	Middle temporal gyrus (L)	64	4.05	−50	−2	−28

The general linear model was used to define clusters of significant group effects (CRPS vs. control) on functional connectivity of each RSN of interest. The brain regions showing significant alterations in functional connectivity at a TFCE-corrected *P* < 0.05 were defined as clusters.

Abbreviations: MNI, Montreal Neurological Institute; CRPS, chronic regional pain syndrome; RSN, resting state network; L, left; R, right; TFCE, threshold-free cluster enhancement.

### Differences in Inter-network Functional Connectivity with the Attention Network

**Figure 2:**
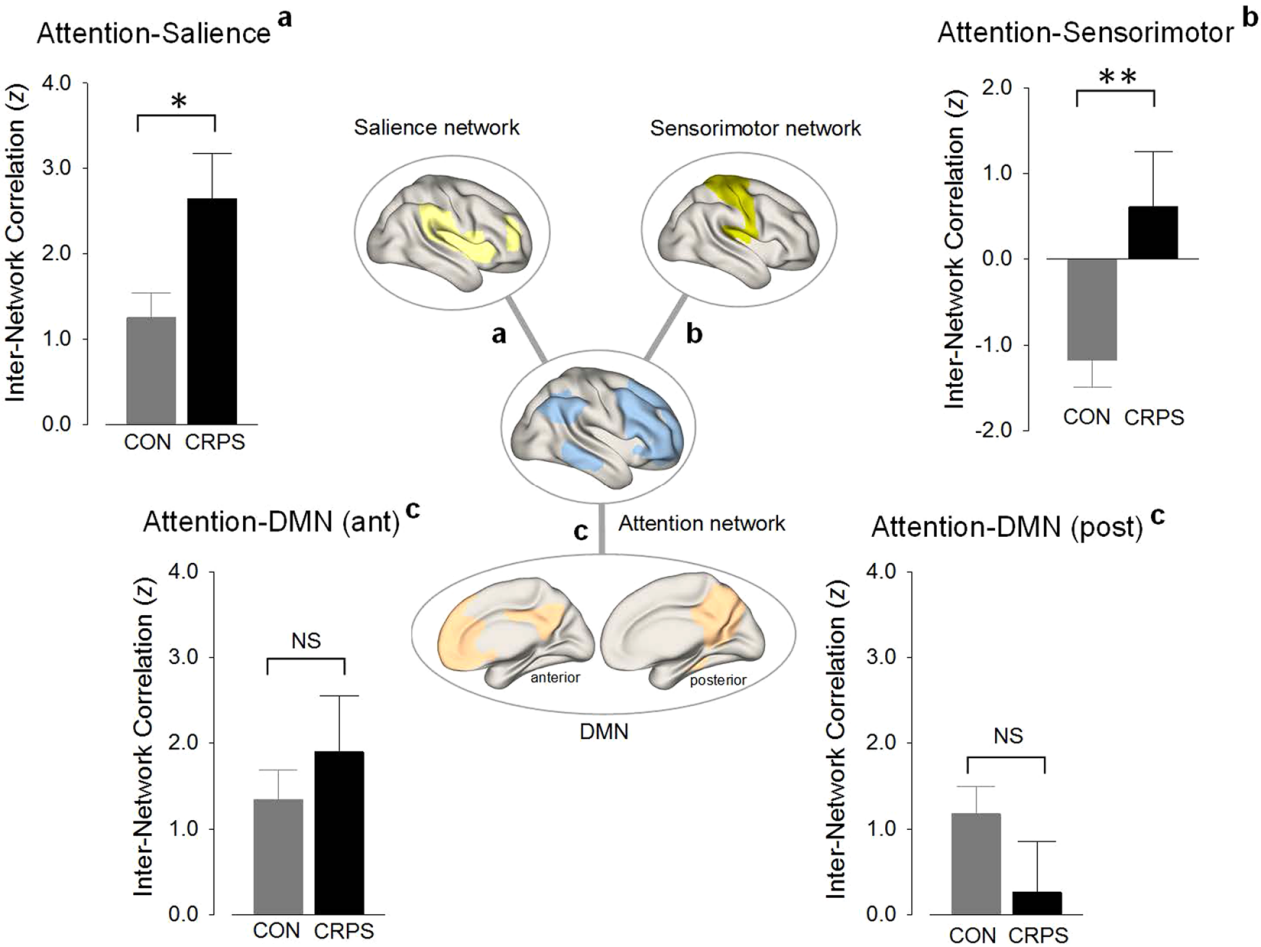
Inter-network correlations between brain networks in the CRPS and control groups. Functional coupling of the attention and salience networks was greater in the CRPS group relative to the control group. Enhanced inter-network connectivity between the attention and sensorimotor networks was also observed in the CRPS group, as compared with the control group. However, there were no significant group differences in the inter-network connectivity between the attention and default mode networks. *Permutation-adjusted *P* < 0.05; **Permutation-adjusted *P* < 0.01; DMN, default mode network; NS, non-significant; CON, control; CRPS, complex regional pain syndrome; ant, anterior; post, posterior.

Group differences in functional connectivities between the attention network and other pain-related RSNs were also determined. Functional coupling between the attention and salience networks was enhanced in individuals with CRPS relative to healthy individuals (permutation adjusted *P* = 0.02, effect size (ES) = 0.63, Fig. 2a). Inter-network connections between the attention and sensorimotor networks were also enhanced in individuals with CRPS relative to healthy individuals (permutation adjusted *P* = 0.004, ES = 0.70) (Fig. 2b). However, there were no significant differences in inter-network connections between the attention and default mode networks (anterior DMN, permutation adjusted *P* = 0.46, ES = 0.21; posterior DMN, permutation adjusted *P* = 0.17, ES = 0.38) (Fig. 2c).

### Relationship between Intra-, Inter-network Functional Connectivity and the Extent of Pain Catastrophizing

**Figure 3:**
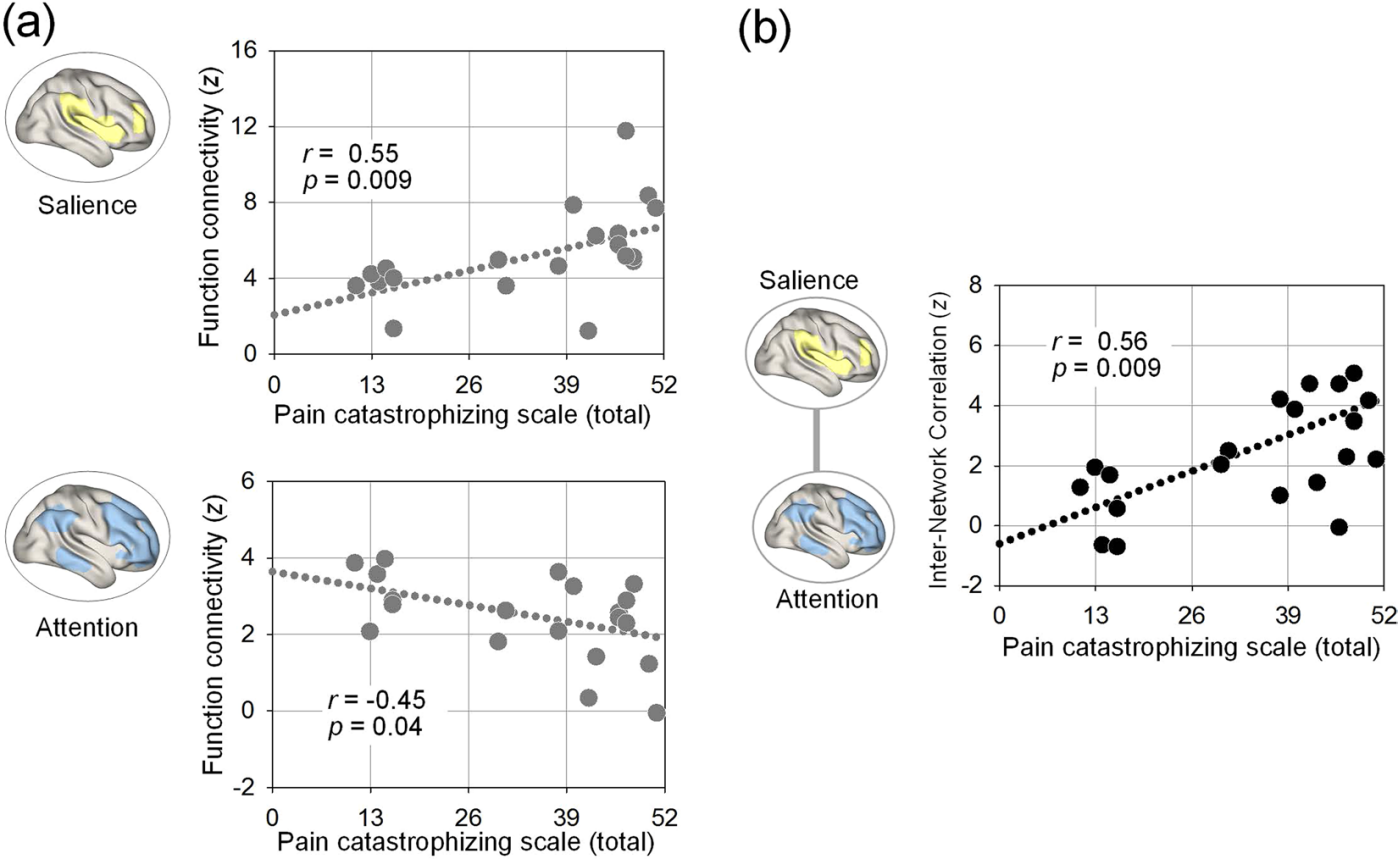
Correlations between the total scores on the Pain Catastrophizing Scale (PCS) in individuals with CRPS and functional connectivity in brain networks. **(a)** Scatter plots and regression lines between PCS scores and intra-network functional connectivity in the salience and attention networks. A significant positive correlation between PCS scores and enhanced intra-network functional connectivity in the salience, while a significant negative correlation between PCS scores and functional connectivity in the attention network were observed in individuals with CRPS. **(b)** Scatter plot and regression line between PCS scores and inter-network functional connectivity. A significant positive correlation was observed between PCS scores and enhanced functional coupling between the attention and salience networks in individuals with CRPS. CRPS, complex regional pain syndrome.

The mean z values were extracted from significant clusters showing between-group differences to examine their relationships with the extent of pain catastrophizing in individuals with CRPS. The PCS scores of individuals with CRPS were positively correlated with functional connectivity of the cluster within the salience network (*r* = 0.55, *P* = 0.009, Fig. 3a). In contrast, higher PCS scores were associated with reduced functional connectivity of the clusters within the attention network (*r* = −0.45, *P* = 0.04, Fig. 3a). Furthermore, there were no significant relationships between the PCS scores and functional connectivity of clusters within the sensorimotor (*r* = 0.11, *P* = 0.63) or the anterior default mode networks (*r* = −0.04, *P* = 0.87).

For the relationships between inter-network functional connectivities and the level of pain catastrophizing, we found that higher PCS scores were associated with closer connections between the attention and salience networks (*r* = 0.56, *P* = 0.009, Fig. 3b). However, PCS scores were not associated with inter-network connections between the attention and sensorimotor networks (*r* = 0.01, *P* = 0.95).

## Discussion

The current study provides one of the first evidence of the role of the attention network and its dynamic interactions with other pain-related RSNs in the case of CRPS. We found significant CRPS-associated alterations in functional connectivities within the attention network as well as other pain-related RSNs such as the salience, sensorimotor, and default mode networks. Furthermore, functional connections between the attention network and both salience and sensorimotor networks were enhanced in individuals with CRPS. These findings may suggest that the attention network may be dynamically involved in the aberrant cognitive process during the perception of pain in CRPS. In addition, a high level of pain catastrophizing in individuals with CRPS was associated with reduced functional connectivity of the attention network, while also being in association with enhanced functional connectivity within the salience network. A higher level of pain catastrophizing was also related to enhanced functional connections between the attention and salience networks.

The attention network, which is also referred to as the frontoparietal or executive attention network, consists of the dorsolateral prefrontal, posterior parietal, and lateral temporal areas^28^. Specifically, the right lateralized attention network has been suggested to be engaged in perception, somesthesis, and pain processing^28^. Given these features of the attention network, its critical role in perception, anticipation, and modulation of pain has been actively studied in healthy population^28–33^. Likewise, in chronic pain conditions such as migraine^34^ and fibromyalgia^32^, aberrant functional connectivity was also observed within the attention network as shown in the present study.

Our study demonstrates novel findings that indicate enhanced functional coupling of the attention network with the salience and sensorimotor networks in individuals with CRPS. Given that pain-related stimuli may elicit functional activation of the salience network^12^ as well as the sensorimotor network^33^, these findings imply that an attentional shift may occur to modify the activation of the salience and sensorimotor networks in response to pain in CRPS. These findings are consistent with those from empirical or model data of network interactions which suggest that a transition occurs in certain brain networks in an effort to address cognitive dysfunctions with a neural basis, such as between the attention, sensorimotor and the salience networks^21,35^.

Interestingly, our findings suggest that the enhanced interaction between the attention and salience networks plays an important role in the process of pain catastrophizing. Pain catastrophizing is frequently observed among individuals with centralized pain condition, and is a detrimental cognitive process characterized by the tendency to interpret pain stimuli in an extremely negative fashion. Individuals who catastrophize pain experience symptoms such as magnified pain intensity and rumination over pain, face difficulties in disengaging from pain^27^. The extent of pain catastrophizing has been known to be related to the intensity of perceived pain and clinical outcomes, including chronicity in several centralized pain conditions^36,37^. Considering that activation of the salience network - which consists of the anterior insula, medial prefrontal cortex, temporoparietal junction, and dorsolateral prefrontal cortex - is sustained while attending to painful stimuli^12^, functional activity within the salience network may be enhanced in individuals with a high level of pain catastrophizing, as shown in the current study. Disrupted functional connectivity within the attention network in CRPS was also found to be associated with high levels of pain catastrophizing, which may indicate impairment in cognitive function in subjects who catastrophize over pain or fixate on pain. Therefore, the positive correlation observed between enhanced inter-network functional connectivity of the attention and salience networks and pain catastrophizing in CRPS patients may indicate the potential involvement of maladaptive pain coping. Likewise, enhanced prefrontal control over the insula, medial thalamus, and periaqueductal gray of the brainstem has been found in other centralized pain conditions^26,38^. Furthermore, reduced intra-network functional connectivity of the attention network in higher levels of pain catastrophizing may support the current interpretation on decompensation of attentional control in CRPS, as catastrophizing pain implies constant fixation and distress with regard to pain, which may render the attention performance less efficient.

It is noteworthy that we also identified CRPS-related functional connectivity alterations in the salience^11,18–20^, default mode^23,26,38^, and sensorimotor networks^20,24,39^, all of which were known to be affected in various conditions regarding centralized pain.

Until now, studies have suggested that brain regions pertaining to the attention and sensorimotor networks may be activated in response to a noxious stimulus^33^. Such findings imply that the sensorimotor network, as opposed to the salience network, may play a dominant role in pain processing under normal condition. However, in the present study, enhanced functional connectivity between the attention and sensorimotor networks was not associated with the level of pain catastrophizing in individuals with CRPS. Considering how catastrophizing pain is a key symptom of centralized pain conditions, it may then be assumed that pain processing in centralized pain conditions may rely more on the dynamic interaction between the attention network and the salience network, rather than the interaction between the attention network and the sensorimotor network^40^.

In addition, the generalizability of our findings to all centralized pain conditions may be limited since the current results were derived from a sample of individuals with CRPS. However, considering that the majority of centralized pain conditions have a shared underlying mechanism^41^, our results may still provide valuable insight on centralized pain conditions in general.

Finally, as inter-network connections were measured using a correlation approach, directional information is lacking. Therefore, future studies will be necessary to determine whether enhanced connections between the attention and salience networks in individuals with CRPS may reflect increased attentional control over the salience network, increased information transfer from the salience network to the attention network, or both.

In conclusion, the present study is the first to characterize alterations in attention network functions and connectivities in response to centralized pain conditions. The enhanced connections between the attention and salience networks along with reduced functional connectivity of the attention network may indicate that altered network connectivity is associated with the maladaptive pain coping which may result from long-standing pain stimuli in individuals with complex regional pain syndrome. Our findings highlight that the two functional networks may be of significance in future research in this area, where improvement in integrative function of the attention network could be a potential target for the treatment of centralized pain conditions, such as, but not exclusive to CRPS.

## Methods

### Participants and Clinical Assessments

The study participants were 21 individuals (16 men, 5 women, mean age 37.7 years ±10.9 standard deviation [SD]) with a diagnosis of CRPS based on the International Association for the Study of Pain criteria (“the Budapest criteria”)^42–44^, and 49 healthy individuals matched for age and sex (39 men, 10 women, mean age 36.8 years ±9.4 SD). Individuals with any axis 1 psychiatric disorders other than depressive disorder, major medical or neurological disorders, a history of traumatic brain injury with loss of consciousness, or any contraindications to MRI were excluded from the study.

For individuals with CRPS, the mean duration of CRPS diagnosis was 27.5 months (range = 1.8–74.5). Detailed information regarding the clinical characteristics of each individual is presented in Table 2. The magnitude and nature of currently experienced pain in individuals with CRPS were assessed using the Visual Analog Scale (VAS) ranging from 0 (no pain) to 10 (the worst imaginable pain), as well as the short-form of the McGill Pain Questionnaire (MPQ)^44^. The rating of pain using the VAS was also collected before and after the MRI scan. All individuals with CRPS also completed the PCS, a 13-item self-administered questionnaire, to assess the degree of catastrophizing behaviors and thoughts about pain. The PCS measures rumination, magnification, and helplessness regarding pain experience.

**Table 2:** Clinical characteristics of participants with CRPS.

CRPS Patient No.	Age	Sex	CRPS duration (months)	Current pain (VAS)	Current pain (MPQ)	CRPS affected side	Inciting trauma
1	43	M	21.1	9	32	Right	Knee ligament injury
2	28	M	4.6	9	40	Left	Operation of toe
3	26	M	74.5	8	41	Right	Spontaneous
4	30	M	48.6	8	34	Right	Strain trauma
5	35	M	20.2	6	27	Left	Fracture of tibia
6	47	M	8.9	8	37	Right	Lumbar disc protrusion
7	40	F	4.0	8	36	Right	Contusion of elbow
8	43	M	35.0	9	44	Right	Fracture of ilium
9	41	F	2.4	8	43	Left	Lumbar disc protrusion
10	44	M	25.5	10	37	Right	Knee ligament injury
11	43	M	72.4	4	34	Left	Cervical disc protrusion
12	21	M	1.8	8	24	Right	Fracture of toe
13	59	F	57.6	8	34	Left	Contusion of foot
14	29	M	11.6	8	34	Left	Spontaneous
15	45	M	21.2	4	15	Right	Fracture of hand
16	36	M	29.3	5	16	Left	Fracture of tibia
17	21	M	7.9	5	22	Right	Fracture of toe
18	59	F	63.2	7	23	Left	Spontaneous
19	30	M	1.9	7	23	Right	Burn
20	45	F	3.7	7	42	Right	Fracture of toe
21	26	M	62.2	5	16	Right	Contusion of elbow

Abbreviations: CRPS, chronic regional pain syndrome; VAS, visual analog scale; MPQ, the short-form of the McGill pain questionnaire.

All participants provided written informed consent to participate in the study. The study protocol was approved by the Institutional Review Board of the Catholic University of Korea College of Medicine, and all procedures were performed in accordance with institutional and national guidelines and regulations.

### Functional MRI Data Acquisition and Processing

All brain imaging data were acquired using a 3.0 Tesla MR scanner (Skyra, Siemens, Erlangen, Germany). Resting-state fMRI data were obtained with a T2*-weighted echo planar imaging sequence using the following parameters: repetition time (TR) = 3,000 ms; echo time (TE) = 20 ms; flip angle (FA) = 90°; field of view (FOV) = 192 mm^2^; slice thickness = 3 mm; 120 volumes; 48 slices. During the resting-state fMRI scan, participants were instructed to keep their eyes closed, not to fall asleep, think of nothing in particular, and let their mind wander freely. For co-registration with the fMRI data, high-resolution T1-weighted structural images were obtained with the following acquisition parameters: TR = 1,900 ms; TE = 2.49 ms; FA = 9°; FOV = 230 mm^2^; slice thickness = 0.9 mm; 208 contiguous sagittal slices.

Functional image data preprocessing was performed using the modules contained within the FMRIB Software Library tools (FSL, http://www.fmrib.ox.ac.uk/fsl). The standard preprocessing steps consisted of motion correction using multi-resolution rigid body co-registration^45^, brain extraction using the FSL Brain Extraction Tool (BET), spatial smoothing with a Gaussian kernel of full width at half maximum of 5 mm, and high-pass filtering at 0.01 Hz. Functional image data of each individual was first co-registered to the corresponding T1-weighted image. These co-registered images were further linearly registered to the Montreal Neurological Institute (MNI) 152 template using affine transformation with 12 degrees of freedom. There were no differences in head motion parameters between the two groups (absolute head motion, the CRPS group 0.145 ± 0.054 mm, the control group, 0.125 ± 0.045, *t* = 1.56, *P* = 0.12; relative head motion, the CRPS group 0.086 ± 0.044 mm, the control group, 0.076 ± 0.032, *t* = 1.01, *P* = 0.32).

Single-subject independent component analysis (ICA) was applied to identify the structural artifacts in each functional image data as implemented in the Multivariate Exploratory Linear Optimized Decomposition into Independent Components (MELODIC)^46,47^. Afterwards, FMRIB’s ICA-based Xnoiseifier (FIX) was used to remove components corresponding to structural artifacts from each functional image data set^48^.

In order to obtain group-level RSNs, group ICA - a model-free and data-driven approach - was implemented to decompose the preprocessed four-dimensional functional images into three-dimensional spatial maps and one-dimensional time series^46,47^. In the current study, functional image data was decomposed into 25 independent components with a temporal concatenation approach. Consequently, 18 components were classified as anatomically and functionally meaningful RSNs corresponding to the functional networks previously described^47^, and 7 components were classified as artifacts by visual inspection of an experienced researcher (S. Y.). Of these 18 identified RSNs, we selected 5 RSNs of interest, which were the salience, sensorimotor, and default mode (anterior and posterior) networks to represent the pain-related RSNs and the right frontoparietal network as the attention network. The abovementioned RSNs of interest were used in subsequent analyses. Component information and spatial maps of all available components, which were thresholded at a level of z = 3.0 (*P* = 0.001) are presented in Supplementary Figure.

A dual regression algorithm was applied to estimate subject-specific time courses and spatial maps, which corresponded to the RSNs of interest derived from the initial group ICA^46^. In the first step of dual regression, the average subject-specific time series of the RSNs of interest was derived using a linear model fit of the RSNs of interest against each individual’s functional data. The second step provided the subject-specific spatial maps for the RSNs of interest, which reflect the degree of synchronization by the temporal regression against each individual’s functional data.

Using the FSLNets (http://fsl.fmrib.ox.ac.uk/fsl/fslwiki/FSLNets), the temporal correlation coefficients between the attention network and pain-related RSNs, including the salience, sensorimotor, and default mode networks were computed based on individual time series of the RSNs. Correlation coefficients between the RSNs, which reflect the strength of inter-network connections, were Fisher z-transformed and were then used for subsequent analyses.

### Statistical Analyses

Demographic characteristics were compared between the two groups using the independent t-test and the chi-square test for continuous variables and categorical variables, respectively.

For the comparison of intra-network functional connectivity, the general linear model (GLM) was applied to examine group effects (CRPS vs. control) on functional connectivities of the RSNs at each voxel level. Age and sex composition were included as relevant covariates. Permutation testing (5,000 permutations) (Nichols *et al*. 2002) with threshold-free cluster enhancement (TFCE) at a significance threshold of *P* < 0.05 was used to perform a family-wise error correction for multiple comparisons^28^. The z values, which represent the functional connectivity of the corresponding RSNs, were extracted from significant clusters at the voxel level based on each subject’s spatial map. The mean z values of significant clusters were then used for further correlation analyses (Fig. 3). Cohen’s *d* was estimated to measure effect size (ES).

For the comparison of inter-network functional connectivity, we used the GLM to examine the group effects (CRPS vs. control) on the inter-network connections between the corresponding RSNs after covarying for age and sex. Permutation-adjusted *P* values with 5,000 permutations of group members (CRPS vs. control) were calculated using a significance threshold of *P* < 0.05^49^.

Pearson correlation analysis was performed to examine the relationships between the total scores of the PCS and intra- or inter-network functional connectivity.

Two-tailed significance of *P* < 0.05 was considered to be statistically significant. Data were analyzed using the Stata SE, v11.0 (Stata Corp, College Station, TX).

All datasets generated and/or analysed during the current study are available from the corresponding author on reasonable request.

## Electronic supplementary material

Supplementary Figure S1
