# Supplementary material for: Altered attentional control over the salience network in complex regional pain syndrome
Source: Sci Rep. 2018 May 10;8:7466. doi: 10.1038/s41598-018-25757-2 (PMC5945627; doi:10.1038/s41598-018-25757-2)
Supplement: Supplementary file 1 — Supplementary Figure S1 [file 41598_2018_25757_MOESM1_ESM.pdf]

Supplementary Information

Title

Altered attentional control over the salience network in complex regional pain syndrome

Short Title

Attention over Salience Network in Pain

Authors

Jungyoon Kim[1,2,+], Ilhyang Kang[1,2,+], Yong-An Chung[3], Tae Suk Kim[4], Eun Namgung[1,2], Suji Lee[1,2], Jin Kyoung Oh[3], Hyeonseok S. Jeong[3], Hanbyul Cho[1], Myeong Ju Kim[1,2], Tammy D. Kim[1,2], Soo Hyun Choi[5], Soo Mee Lim[6] , In Kyoon Lyoo[1,2,7,*], and Sujung Yoon[1,2,*]

Affiliations

[1] Ewha Brain Institute, Ewha W. University, Seoul, South Korea

[2] Department of Brain and Cognitive Sciences, Ewha W. University, Seoul, South Korea

[3] Department of Radiology, Incheon St. Mary's Hospital, The Catholic University of Korea College of Medicine, Seoul, South Korea

[4] Department of Psychiatry, The Catholic University of Korea College of Medicine, Seoul, South Korea

[5] School of Science and Engineering, Tulane University, New Orleans, USA

[6] Department of Radiology, Ewha W. University College of Medicine, Seoul, South Korea

[7] College of Pharmacy, Graduate School of Pharmaceutical Sciences, Ewha W. University, Seoul, South Korea

[+]These authors contributed equally to this study.

*correspondence and requests for materials should be addressed to I.K.L (email: inkylyoo@ewha.ac.kr) or S.Y. (email: sujungjyoon@ewha.ac.kr)

Corresponding Authors

In Kyoon Lyoo, MD, PhD
Ewha Brain Institute, Department of Brain and Cognitive Sciences, and Graduate School of Pharmaceutical Sciences, Ewha W. University, 52 Ewhayeodae-gil, Seodaemun-gu, Seoul 03760, South Korea.
Email: inkylyoo@ewha.ac.kr, Phone: +82-2-3277-6550, FAX: +82-2-3277-6562

Sujung Yoon, MD, PhD
Ewha Brain Institute and Department of Brain and Cognitive Sciences. Ewha W. University, 52 Ewhayeodae-gil, Seodaemun-gu, Seoul 03760, South Korea.
Email: sujungjyoon@ewha.ac.kr, Phone: +82-2-3277-2478, FAX: +82-2-3277-6562

Supplementary Figure

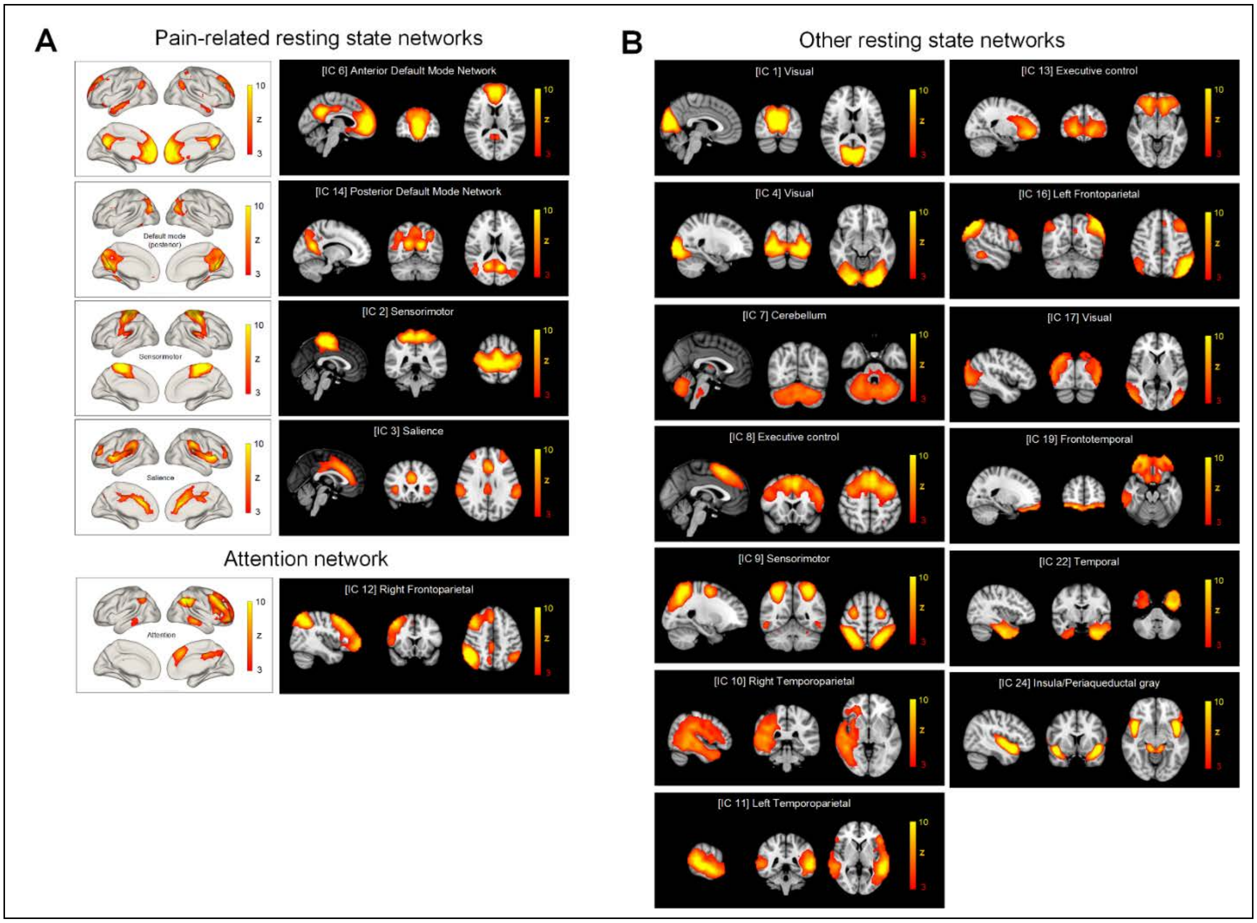

Supplementary Figure S1. Among 25 independent components (ICs) based on the group ICA of the total participants (n = 70), five RSNs of interest which are the attention, salience, sensorimotor, default mode (anterior), and default mode (posterior) networks were selected (A). Spatial maps were converted to z score images and were thresholded at z = 3.0 (*P* = 0.001). The number of IC indicates the amount of variance explained by the corresponding IC in decreasing order. Apart from the five selected RSNs of interest, other ICs which demonstrated considerable correspondence to the major RSNs are presented in panel B.

ICA, independent component analysis; RSN, resting-state network.
